# Operative choice for subtrochanteric femoral fracture in school-aged children: Triple elastic stable intramedullary nail versus locking plate

**DOI:** 10.3389/fped.2022.894262

**Published:** 2022-07-26

**Authors:** Pan Hong, Xiaolong Zhao, Renhao Ze, Saroj Rai, Ruikang Liu, Jin Li, Xin Tang

**Affiliations:** ^1^Department of Orthopaedic Surgery, Union Hospital, Tongji Medical College, Huazhong University of Science and Technology, Wuhan, China; ^2^Department of Orthopaedics, First Hospital of Wuhan, Tongji Medical College, Huazhong University of Science and Technology, Wuhan, China; ^3^Department of Orthopaedics and Trauma Surgery, Blue Cross Hospital, Tripureswor, Kathmandu, Nepal; ^4^Department of Orthopaedics and Trauma Surgery, Karama Medical Center, Dubai Investment Park Br, Dubai, United Arab Emirates; ^5^Department of Endocrinology, Union Hospital, Tongji Medical College, Huazhong University of Science and Technology, Wuhan, China

**Keywords:** subtrochanteric femoral fracture, children, triple elastic stable intramedullary nail, locking plate, retrospective study

## Abstract

**Background:**

The management strategy of subtrochanteric fractures remains controversial, and triple elastic stable intramedullary nail (ESIN) has not been reported for pediatric subtrochanteric fractures. This study aimed to compare the clinical effects of treating school-aged children with subtrochanteric fractures with triple ESINs versus locking plates.

**Methods:**

We conducted a retrospective review of pediatric patients with subtrochanteric femoral fracture receiving either triple ESINs (TE) or locking plates (LPs) between January 2010 and January 2018. Sixteen patients in each group with matched age, sex, and fracture characteristics were included in the study. The preoperative data, including baseline information of the patients, fracture pattern, and types of surgical procedure, were collected from the hospital database. Patients were followed-up at the outpatient clinic in the 3rd month, 6th month, 12th month, and annually afterward. Hardware removal was performed at 9 – 18 months after the primary surgery.

**Results:**

In all, 16 patients (8.4 ± 1.5-year-old, 7 boys, 9 girls) in the TE group and 16 patients (8.4 ± 1.4-year-old, 7 boys, 9 girls) in the LP group were included. There was significantly less operative time, reduced estimated blood loss, and shortened hospital stay for the TE as compared with the LP (*P* < 0.001). However, higher fluoroscopy frequency was observed in the TE group than in the LP group (*P* < 0.001). The time to union was faster in the TE group than in the LP group (P = 0.031). However, the angulation was higher in the TE group (3.2 ± 0.6) than the LP group (1.8 ± 0.5), and the incidence of implant prominence was higher in the TE group (7/16, 43.8%) than the LP group (1/16, 6.3%).

**Conclusion:**

Compared with the locking plates, triple ESINs demonstrated significantly less operative time, reduced estimated blood loss, and shortened hospital stay. Besides, both TE and LP groups produced satisfactory outcomes in school-aged children with subtrochanteric fractures. Therefore, TE remains a feasible choice for subtrochanteric fractures in school-aged children.

## Background

Subtrochanteric fractures in children are rare, and most injuries result from high-energy trauma (1). The management strategy for this injury remains controversial (1, 2). Simple spica casting for younger children, closed reduction with elastic stable intramedullary nail (ESIN), rigid nail fixation for adolescents, open reduction and plate fixation, and external fixator (EF) have been reported for this condition (3–6).

Elastic stable intramedullary nail (ESIN) is widely used in school-aged children with long bone diaphyseal fractures, and it has been reported for children with subtrochanteric fractures (4, 6–8). However, compared with plating, its superiority remains controversial (9, 10). In these studies, double nails were adopted to treat this condition. Triple ESINs have not been reported for pediatric subtrochanteric fractures, but triple ESINs have been employed in children with unicameral bone cysts in the proximal femur with pathological fractures (11). We believe that Triple ESINs could provide better support and stability at the fracture site, especially in pathological fractures with a thinner cortex and a larger diameter. Compared with plating with longer incisions and difficult removal in secondary surgery, ESIN seems a promising choice for children with subtrochanteric fractures. Therefore, triple ESINs have been employed at our institute for this condition since 2014. This study aimed to compare the clinical effects of school-aged children with subtrochanteric fractures treated with triple ESINs versus locking plates. We propose the hypothesis that triple ESIN delivers better clinical outcomes than locking plates for school-aged children with subtrochanteric fractures.

## Methods

**FIGURE 1:**
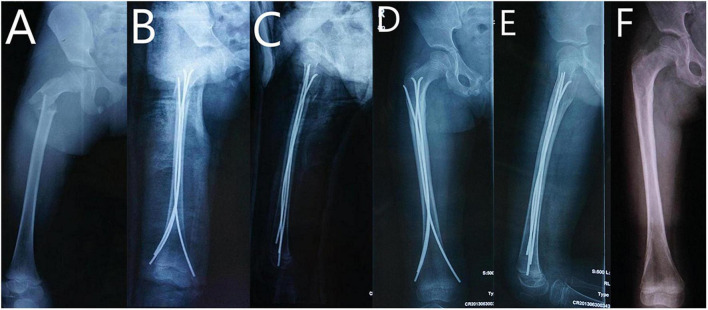
9 year-old girl with right subtrochanteric fracture treated with triple ESINs. **(A)** AP view of femur before surgery. **(B)** AP view of femur after surgery. **(C)** Lateral view of femur after surgery. **(D)** AP view of femur at 6th month follow-up. **(E)** Lateral view of femur at 6th month follow-up. **(F)** AP view of femur after implant removal.

**FIGURE 2:**
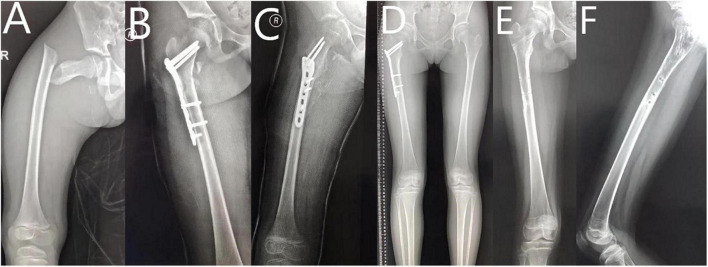
10 year-old girl with right subtrochanteric fracture treated with locking plate. **(A)** AP view of femur before surgery. **(B)** AP view of femur after surgery. **(C)** Lateral view of femur after surgery. **(D)** AP view of femur at 6th month follow-up. **(E)** AP view of femur after implant removal. **(F)** Lateral view of femur after implant removal.

A retrospective review of pediatric patients with subtrochanteric femoral fracture receiving either triple ESINs (TE, see Figure 1) or locking plates (LP, see Figure 2) was conducted between January 2010 and January 2018. Sixteen patients in each group with matched age, sex, and fracture characteristics were included (Table 1). The preoperative data, including baseline information of the patients, fracture pattern, and types of surgical procedure, were collected from the hospital database.

**TABLE 1:** Parameters of patients in the TE and LP group.

Variables	TE (*N* = 16)	LP (*N* = 16)	*P*-value
Age(year)	8.4 ± 1.5	8.4 ± 1.4	0.743
Body weight (Kg)	35.8 ± 1.0	36.2 ± 1.3	0.292
Sex			>0.999
Female	9	9	
Male	7	7	
Seinsheimer classification			>0.999
I	2	2	
IIA	4	4	
IIB	7	7	
IIC	3	3	
Cause of injury			0.708
Falling	11	10	
Vehicle accident	5	6	

The inclusion criteria were as follows: (1) 6–14 years old at the time of fracture; (2) subtrochanteric femoral fracture without concomitant injury in the ipsilateral lower extremity; and (3) a follow-up period of more than 24 months. The exclusion criteria comprised the following: (1) patients aged more than 14 years; (2) severely comminuted fractures; (3) fractures associated with neurovascular injuries; (4) pathological fractures and open fractures; (5) patients treated with double ESINs; and (6) body weight over 50 kg.

Subtrochanteric fracture is defined as the percentage of the distance between the lesser trochanter and fracture line/the length of the femur that is less than 10% (4). Seinsheimer fracture classification was adopted to evaluate the injuries (12). Limb length discrepancy (LLD) and angulation were evaluated according to radiographs, and Hip function was assessed using the Harris Hip Score scale (13). Hardware removal was performed at 9-18 months after the primary surgery.

Primary outcomes included operating time, hospital stay, and estimated blood loss. Secondary outcomes included frequency of fluoroscopy, time to union, length of limb discrepancy (LLD), Harris score at last follow-up, and complications. Loss of reduction, non-union, deep infection, and angulation of more than 10 degrees (in the coronal or sagittal plane) were classified as major complications in this study.

### Surgical technique of triple ESINs

The diameter of ESIN was determined as 1/3–2/5 of the diameter of the narrowest site of the femoral shaft. After retrograde insertion of double ESINs, anteroposterior (AP) and lateral views were required to confirm the alignment and reduction. Afterward, Patrick (FABER [Flexion, Abduction, and External Rotation]) test was performed to evaluate the stability. If the fracture site demonstrated >1 cm displacement or translation or >15 degrees angulation, a third ESIN was inserted from the lateral incision of the distal femur. The diameter of the third ESIN was the same as the earlier choice. Afterward, the Patrick test was performed again to evaluate the stability.

The surgeon in charge assessed the patient’s recovery during the follow-ups. Spica casting or brace was used for 4 weeks after the surgery. Then, all patients were followed-up at the outpatient clinic in the 3rd month, 6th month, and 12th month. Afterward, annual follow-ups were recommended. X-ray evaluation and physical examination were conducted at every follow-up.

Four senior surgeons with more than 5 years of working experience in Pediatric Orthopedics performed these operations in this study. Besides, all of them could perform triple ESIN and LP proficiently.

### Statistical analysis

Appropriate statistical methods were used for descriptive statistics. Continuous data were compared using an independent sample *t-*test, and categorical variables were tested by Fisher’s exact test. All statistical tests were two-tailed. Statistical analysis was performed using the IBM SPSS statistics version 20 software (SPSS Inc., Chicago, Illinois).

## Results

As shown in Table 1, 16 patients (8.4 ± 1.5 years old, 7 boys, 9 girls) in the TE group and 16 patients (8.4 ± 1.4-year-old, 7 boys, 9 girls) in the LP group were included. Patients in both groups were followed up for more than 24 months. There was no significant difference between the two groups concerning the demographic parameters, including sex, age, weight, Seinsheimer classification, and cause of injury.

Comparing clinical parameters (Table 2), there was significantly less operative time, reduced estimated blood loss (EBL), and shortened hospital stay for the TE as compared with the LP (*P* < 0.001). However, higher fluoroscopy frequency was observed in the TE group than in the LP group (*P* < 0.001). The time to union was also faster in the TE group than in the LP group (P = 0.031). Regarding LLD and Harris function scores, there was no significant difference between the two groups.

**TABLE 2:** Clinical parameters of the TE and the LP group.

Variables	TE (*N* = 16)	LP (*N* = 16)	*P*-value
Operating time (min)	50.0 ± 9.00	76.5 ± 8.50	<0.001*
Hospital stay (day)	3.3 ± 1.1	4.8 ± 1.2	<0.001*
Estimated blood loss (ml)	12.1 ± 5.9	74.4 ± 6.3	<0.001*
Fluoroscopy (times)	18 ± 5.9	10 ± 2.9	<0.001*
Time to union (week)	11.8 ± 2.0	13.2 ± 1.7	0.031
Limb length discrepancy (cm)	0.2 ± 0.1	0.2 ± 0.1	0.405
Harris score at last follow-up	93.7 ± 1.9	94.4 ± 2.0	0.332

*P value ≤ 0.05.

As shown in Table 3, there was no patient with loss of reduction, non-union, and deep infection in either group. The angulation was significantly higher in the TE group (3.2 ± 0.6) than in the LP group (1.8 ± 0.5) (*P* < 0.001), but the angulation in the TE group was less than 5 degrees. The incidence of implant prominence was significantly higher in the TE group (7/16, 43.8%) than in the LP group (1/16, 6.3%) (*P* < 0.001).

**TABLE 3:** Complications after surgery.

Complication	TE (*N* = 16)	LP (*N* = 16)	*P*-value
Loss of reduction	0	0	>0.999
Non-union	0	0	>0.999
Implant prominence	7 (43.8%)	1 (6.3%)	<0.001*
Angulation (degrees)	3.2 ± 0.6	1.8 ± 0.5	<0.001*
Superficial infection	1 (6.3%)	1 (6.3%)	>0.999
Deep infection	0	0	>0.999

*P value ≤ 0.05.

## Discussion

Triple ESINs proved to be a minimally invasive approach for school-aged children with subtrochanteric fractures. Shorter operative time, length of hospital stay, and comparable clinical outcomes to LP can be achieved with triple ESINs. In the TE group, the anatomic reduction was unnecessary, and patients did not suffer from the longer incision and increased blood loss. Therefore, patients in the TE group resulted in better primary outcomes accordingly.

Subtrochanteric fracture is rare and usually results from high-energy injury (14). So far, various treatments have been reported, including simple spica casting for younger children and surgeries with internal or external fixation for older children (2). Spica casting following traction requires a prolonged hospital stay and demonstrates the limited capability of restoring the limb length and alignment (3). EF is an alternative for patients with polytrauma or open injuries, but the limited region for pin placement in the proximal femur and difficulty of daily pin care leads to the waning enthusiasm for EF (15). ESIN has been widely applied in treating femoral diaphyseal fractures, but more complications were reported in patients with proximal femoral shaft fractures treated with double ESINs (16). For decades, LP has been reported for this condition and delivers anatomic reduction and satisfactory clinical outcomes (9, 10). However, it requires a longer incision, and refracture after plate removal can also be a nuisance.

Numerous comparative studies reported on the application of ESIN versus plate in the treatment of diaphyseal long bone fracture (17, 18). In length stable fracture, ESIN seems a better choice (9, 19). As for length unstable or comminuted fracture, the plate provides better stiffness and stability (4, 19). Parikh et al. reported elastic nailing represented an important option for difficult-to-manage femur fractures (7). A comparative study reported by Li et al. in 2013 demonstrated better outcome scores and a lower overall complication rate in plate fixation versus titanium elastic nails (9). Xu et al. reported a similar comparative study in 2018, and it demonstrated plate fixation as a more rigid fixation associated with a lower complication rate (10). In the aforementioned studies, double nails were adopted. In contrast, some authors espoused the applications of double nails for pediatric subtrochanteric fractures with satisfactory outcomes (6–8, 20). Certain techniques, including the placement of nails in the femoral neck or the nail’s perforation of the femoral neck cortex, were recommended in these articles (8, 20).

At our institute, double and triple nails were adopted for this condition. Alignment, fracture displacement, and stability were assessed during the surgery to decide the necessity of a third nail. If the fracture site demonstrated > 1 cm displacement or translation or > 15 degrees angulation, a third ESIN was inserted from the lateral incision of the distal femur. Besides, in our study, there was no patient with malunion or severe angulation in the group of TE, consistent with the previous study (8, 20).

Triple ESINs have been reported for pediatric patients with pathological fractures in the proximal femur (11). Besides, triple nails and a quartet of nails have been reported for challenging femoral shaft fractures in children (21, 22). Therefore, triple nails are feasible choices for pediatric subtrochanteric fractures. To overcome the resistance when passing through the narrow site, the ESIN is required to push patiently.

To the best of our knowledge, our investigation is the first comparative study of triple nails vs. LPs for school-aged children with subtrochanteric fractures. The result shows that the angulation is higher in the TE group but still less than 5 degrees. Both TE and LPs delivered satisfactory clinical outcomes, with minimal angulation and an excellent Harris score. However, shorter operative times and hospital stays were observed in the TE group because of the minimally invasive nature of nailing. The incidence of implant prominence was higher in the TE group because the end of the nail was left around 1-2 cm above the cortex for easier removal at our institute. However, the implant prominence is mostly tolerable, and the Harris score was excellent. Patients in the TE group healed faster in the TE group partly because of its minimally invasive nature and limited stripping of the periosteum around the fracture site. With a shorter incision, the blood loss was reduced in the TE group compared with the LP group.

There were several limitations in our study. First, it was a retrospective study with a small sample size; therefore, our findings should be interpreted with caution. Second, the allocation of patients to the TE group or the LP group partly depended on the surgeon’s preference, and this strategy may cause allocation bias. Third, the follow-up in our study was not long enough to investigate the long-term influence on skeletal growth. Lastly, this study did not include patients receiving double nails and simple spica casting. Further study of biomechanics is required to validate the increased stiffness and stability of the third ESIN and compare its features against LPs.

## Conclusion

Compared with locking plates, triple ESINs demonstrated significantly less operative time, reduced estimated blood loss, and shortened hospital stay. Besides, both TE and LP groups produce satisfactory outcomes in school-aged children with subtrochanteric fractures. Therefore, TE remains a feasible choice for subtrochanteric fractures in school-aged children.

## Data availability statement

The original contributions presented in the study are included in the article/supplementary material, further inquiries can be directed to the corresponding author/s.

## Ethics statement

The studies involving human participants were reviewed and approved by Ethics Committee of Tongji Medical College, Huazhong University of Science and Technology (IORG No: IORG0003571). Written informed consent to participate in this study was provided by the participants’ legal guardian/next of kin. Written informed consent was obtained from the individual(s), and minor(s)’ legal guardian/next of kin, for the publication of any potentially identifiable images or data included in this article.

## Author contributions

JL and XT were in charge of the main idea and is the guarantor of integrity of the entire clinical study. PH and XZ were in charge of the study concepts, design, and manuscript preparation and editing. RL and SR were in charge of the language polishing and the grammar revision. RZ and PH were in charge of the collection of the data. RL performed the statistical analysis. All authors read and approved the final manuscript.

## Abbreviations

ESIN: triple elastic stable intramedullary nail
TE: triple ESINs
LP: locking plate
EF: external fixator
LLD: Limb length discrepancy
EBL: estimated blood loss.
